# Antibiotic Sensitivity of *Proteus mirabilis* Urinary Tract Infection in Patients with Urinary Calculi

**DOI:** 10.1155/2022/7273627

**Published:** 2022-12-21

**Authors:** Licai Mo, Jiajia Wang, Jiao Qian, Minfei Peng

**Affiliations:** ^1^Department of Urology, Taizhou Hospital of Zhejiang Province Affiliated to Wenzhou Medical University, Linhai, Taizhou 317000, Zhejiang, China; ^2^Department of Traditional Chinese Medicine, Taizhou Hospital of Zhejiang Province Affiliated to Wenzhou Medical University, Linhai, Taizhou 317000, Zhejiang, China; ^3^Department of Clinical Laboratory, Taizhou Hospital of Zhejiang Province Affiliated to Wenzhou Medical University, Linhai, Taizhou 317000, Zhejiang, China

## Abstract

**Background:**

The study's objective was to determine *Proteus mirabilis* susceptibility in individuals with urinary tract infections and stones to antibiotics and prescribe optimal antimicrobial treatment.

**Methods:**

Nonrepetitive *Proteus mirabilis* strains were isolated from urine specimens obtained from 317 patients diagnosed with urinary stones from January, 2018, to December, 2021. A VITEK mass spectrometer was used for species identification, and a VITEK-compact 2 automatic microbial system was used for the antimicrobial susceptibility test (AST). Susceptibility to imipenem and cefoperazone/sodium sulbactam was tested by the disc diffusion method (K-B method). The antibiotic sensitivity of the strains was analyzed by sex and season.

**Results:**

A total of 317 patients were reviewed: 202 females (63.7%) and 115 males (36.3%). *Proteus mirabilis* infections were observed during spring (21.8%, *n* = 69), summer (26.2%, *n* = 83), autumn (33.8%, *n* = 107), and winter (18.2%, *n* = 57). *Proteus mirabilis* infections in females were diagnosed most often during the fall (24.3%, *n* = 77) and during the summer in males (11.0%, *n* = 35) (*p* = 0.010). Female patients responded best to levofloxacin (*p* = 0.014), and male patients responded best to sulfamethoxazole (*p* = 0.023). Seasonal variation in antibiotic sensitivity was confirmed, with significantly higher rates in the winter for cefuroxime (*p* = 0.002) and sulfamethoxazole (*p* = 0.002). Significant seasonal increases were also found in levofloxacin sensitivity during the summer (*p* = 0.005).

**Conclusions:**

Highly effective antibiotics such as cefoxitin and ceftazidime should be used empirically by considering antibiotic sensitivity changes by sex, season, and year. Regional studies should be conducted frequently.

## 1. Introduction

Bacterial resistance is one of the biggest risks to global public health since it is growing more prevalent [1, 2]. Urinary tract infections (UTIs) should be taken seriously. Urolithiasis is the most common urological disease. A high percentage of patients with urinary stones develop UTIs, and the two are closely linked. In addition, some infected stones may contain bacteria. *Proteus mirabilis*, a member of the *Enterobacteriaceae* family, can cause UTIs and is the second leading pathogen after *Escherichia coli* [3, 4]. *Proteus mirabilis* is closely associated with complicated UTIs, especially in patients with functional or anatomical abnormalities, such as urinary stones and long-term catheters [5].

The abuse of antibiotics has led to enhanced bacterial pathogenicity due to resistance [6]. The resistance of enterobacteria, including *Proteus mirabilis*, has increased significantly, particularly to cephalosporins, which poses a serious challenge for the clinical treatment of UTIs [7]. The features and sensitivity patterns of urine bacteriology in patients with stones and *Proteus mirabilis* infection have not been fully researched, particularly in China. Therefore, we conducted this study to investigate the characteristics of *Proteus mirabilis* associated with UTIs in patients with urinary stones and to guide the correct and effective clinical treatment.

## 2. Methods

### 2.1. Study Background and Population

This was a retrospective analysis of patients with urinary stones and *Proteus mirabilis* infection who visited Taizhou Hospital of Zhejiang Province affiliated to Wenzhou Medical University, a 1500-bed medical center located in Taizhou, Zhejiang Province, from January, 2018, to December, 2021. Two infectious disease specialists reviewed the urine culture and antimicrobial susceptibility test results. Two clinicians collected the following medical records: diagnosis, admission date, age, sex, urine culture results, and in vitro antimicrobial susceptibility. Inclusion criteria were as follows: (1) nonenhanced CT was employed to diagnose urinary calculi. (2) Only patients with stones in which *Proteus mirabilis* was identified as a urogenic pathogen were eligible. (3) The first *Proteus mirabilis* isolated from the urine of each stone patient was considered. Exclusion criteria were as follows: patients with multiple positive urine cultures were excluded. The study protocol was approved by the Medical Ethics Committee of Taizhou Hospital, Zhejiang Province. The seasons were divided into spring (March-May), summer (June-August), autumn (September-November), and winter (December-February of the following year).

### 2.2. Collection of Urine Specimens

After routine perineal disinfection of the patient, approximately, 25 ml of urine was collected from early morning to mid-morning and placed in a sterile cup. The specimen was sent for examination immediately after collection. If testing could not be performed within 30 minutes of collection, the specimen was stored in a 4°C refrigerator for no more than 24 hours.

### 2.3. Reagents and Instruments

A VITEK mass spectrometer, VITEK-compact2 automatic microbial system, and supporting antimicrobial sensitivity card GN334 were purchased from bioMerieux, France. Antimicrobial-sensitive discs of imipenem and cefoperazone/sodium sulbactam were purchased from Oxiod, UK.

### 2.4. Quality Control Strain

*Escherichia coli* (ATCC 25922) and *Pseudomonas aeruginosa* (ATCC 27853) were purchased from Guangzhou Dijing Microbiological Technology Co., Ltd. (Guangzhou, Guangdong, China) and used for quality control during the AST.

### 2.5. Antimicrobial Sensitivity Test

Sensitivity to levofloxacin, cefuroxime sodium, cefoxitin, ceftazidime, ceftriaxone, cefoperazone/sulbactam, cefepime, amicacin, piperacillin/tazobactam, and sulfamethoxazole was evaluated by a VITEK-compact2 automatic microbial system with supporting reagents. The K-B method was used to evaluate sensitivity to imipenem and cefoperazone/sulbactam, and the antimicrobial sensitivity test results were measured according to the guidelines of the CLSI M100 31TH standard of 2021 [8].  Table 1 summarizes the antimicrobials used to assess the resistance/sensitivity pattern of isolated *Proteus mirabilis* strains.

### 2.6. Statistical Analysis

Data analysis was carried out with the Statistical Package for Social Sciences (SPSS Inc., Chicago, IL, USA) 25.0 for Windows. The data are presented as the mean ± standard deviation and percentages. A chi-square test was used to detect sex and seasonal differences in the uropathogenicity of *Proteus mirabilis*. When a Chi-square test was not suitable, Fisher's exact probability test was used. The significant *p* level established was <0.05.

### 2.7. Ethical Approval

The research followed the ethical guidelines set out in the 1964 Declaration of Helsinki and its subsequent revisions and was approved by the local ethics committee (Medical Ethics Committee of Taizhou Hospital, Zhejiang Province, China, K20220305), and informed consent was waived.

## 3. Results

### 3.1. Basic Characteristics of the Patients

**Figure 1:**
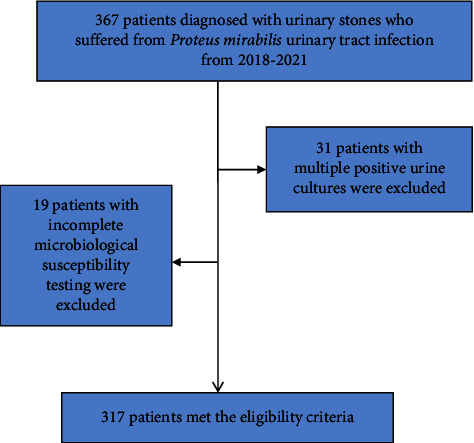
Flowchart for the selection of patients diagnosed with urinary stones who suffered from *Proteus mirabilis* urinary tract infection in this study.

**Figure 2:**
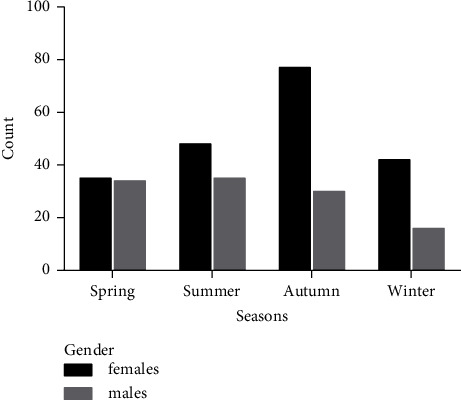
The distribution of *Proteus mirabilis* according to season and sex.

A total of 317 patients (59.9 ± 18.4 years old) with urinary calculi combined with midcourse urine culture suggesting *Proteus mirabilis* infection were included (Figure 1). A total of 202 (63.7%) were females (57.3 ± 19.7 years old) and 115 (36.3%) were males (64.6 ± 14.7 years old). The prevalence of *Proteus mirabilis* infection was 21.8% (*n* = 69) during spring, 26.2% (*n* = 83) during summer, 33.8% (*n* = 107) during autumn, and 18.2% (*n* = 58) during winter. In females, the infection rate was 24.3% (*n* = 77) during autumn and 11.0% (*n* = 35) during spring, and in males, *Proteus mirabilis* infection was the most common during summer (11.0%, *n* = 35) and the rarest during winter (5.1%, *n* = 16) (*p* = 0.010, Figure 2).

### 3.2. Sex Distribution of Antibiotic Sensitivity

According to CLSI guidelines, all 317 strains of *Proteus mirabilis* cultured from patients with urinary calculi were submitted for routine antibiotic sensitivity tests.  Table 2 lists the differences in the sex distribution of *Proteus mirabilis* strains when treated with 12 different antibiotics. Overall, the distribution of susceptibility to the tested antibiotics (meropenem, imipenem, cefuroxime sodium, cefoxitin, ceftazidime, ceftriaxone, cefoperazone/sulbactam, cefepime, amikacin, and piperacillin/tazobactam) was the same in both male and female patients. However, female patients were more responsive to levofloxacin (62.9% vs. 48.7%, *p* = 0.014), and male patients were more responsive to sulfamethoxazole (41.7% vs. 29.2%, *p* = 0.023).

### 3.3. Seasonal and Annual Prevalence of Antibiotic Sensitivity

Table 3 lists the annual and total susceptibility rates of *Proteus mirabilis* strains to 12 different antibiotics. Overall, the sensitivity rates of the tested antibiotics showed a relatively stable pattern over the approximately three-year study period, although the annual sensitivity rates of levofloxacin in 2021 (54.8%) were higher than those in 2020 (50.8%) (*p* = 0.028), and the annual sensitivity rates of cefuroxime decreased each year from 58.4% in 2019 to 52.7% in 2020 to 41.4% in 2021 (*p* = 0.045). The total sensitivity of *Proteus mirabilis* strains to antibiotics was the highest for amikacin (99.1%), meropenem (98.1%), imipenem (96.2%), cefoperazone/sulbactam (95.9%), and piperacillin-tazobactam (87.7%) (Table 3). In contrast, sulfamethoxazole was the least effective antibiotic, with only 33.8% of *Proteus mirabilis* isolates showing sensitivity. The overall three-year rates sensitivity to levofloxacin (57.7%), cefuroxime (50.8%), and ceftriaxone (61.8%) ranged from 50 to 65%. In general, the rate of *Proteus mirabilis* sensitivity to cephalosporin was higher than 50%, and the rate of sensitivity to cefoperazone/sulbactam was the highest (96.9%) (third-generation cephalosporin + *β*-lactamase inhibitor). Seasonal variations in antibiotic susceptibility rates were confirmed. There was a significant seasonal increase in sensitivity to cefuroxime (65.5%, *p* = 0.002) and sulfamethoxazole (48.3%, *p* = 0.002) during winter (December to February). Summer (June-August) was associated with a higher sensitivity to levofloxacin than other seasons (72.3%, *p* = 0.005). The rates of sensitivity to meropenem, imipenem, cefoxitin, ceftazidime, ceftriaxone, cefoperazone, sulbactam, cefepime, amikacin, and piperacillin-tazobactam among *Proteus mirabilis* strains isolated from urinary stones showed no significant difference when stratified by season (Table 4).

## 4. Discussion

*Proteus mirabilis* is a Gram-negativerod-shaped bacterium that frequently causes catheter-associated UTIs that may be associated with urolithiasis due to the biofilm-forming ability and invasion of urinary epithelial cells by urease, which catalyzes the hydrolysis of urea leading to alkalinization of urine and the development of bladder or kidney stones [5]. In this regard, *Proteus mirabilis* is the leading cause of struvite formation with magnesium ammonium phosphate and carbonate apatite [9]. UTIs caused by *Proteus mirabilis* are generally more severe than those caused by *E. coli* and are associated with a higher incidence of pyelonephritis [10]. To our knowledge, the characteristics and antibiotic-sensitive patterns of *Proteus mirabilis* infection in kidney stone patients have not been extensively studied thus far, especially in China; thus, an in-depth understanding of the patterns of antibiotic sensitivity in *Proteus mirabilis* is necessary to ensure effective treatment. Therefore, we conducted this study to investigate the characteristics of *Proteus mirabilis* sensitivity to antibiotics in patients with urinary calculi and to provide evidence for appropriate antimicrobial therapy. *Proteus mirabilis* may exhibit different epidemiological characteristics due to season, sex, age, and regional differences. Therefore, regional studies conducted during different seasons are essential for a better understanding of the disease, effective treatment, and prevention of complications. Our review found that *Proteus mirabilis* infection is more common in women than men as are infective struvite stones [11]. There have been few studies on the susceptibility of *Proteus mirabilis* to antibiotics associated with urinary tract infection or urinary stones, particularly during various seasons. UTIs caused by *E. coli* or *Klebsiella* are more common during summer and less common during spring [12]. Our study found that UTIs caused by *Proteus mirabilis* in urolithiasis patients were the most common during autumn and the least common during winter. If sex was considered, *Proteus mirabilis* infection in females was more common during autumn and less common during spring. *Proteus mirabilis* infection in males was more common during summer and less common during winter. Therefore, sex, season, and etiological factors should be considered before initiating empiric treatment in light of these findings.

Understanding antibiotic sensitivity in the region where patients live will help in selecting the appropriate empiric antibiotic for the treatment of *Proteus mirabilis* infection. Over time, however, antibiotic sensitivity patterns in the region are likely to change. Especially in developing countries such as China, antibiotic sensitivity rates are quite low due to the inappropriate use of antibiotics. In a study conducted from 2010 to 2015 in China, the most effective antibiotics for patients with *Proteus mirabilis* infection and urinary calculi were as follows: furantoin (0.6%), tigecycline (15.4%), sulfamethoxazole (51%), ampicillin (56.5%), cefazolin (55.8%), imipenem (93.4%), meropenem (100%), ciprofloxacin (67.6%), levofloxacin (85.6%), cefoxitin (93.7%), cefoperazone sulbactam (100%), ceftriaxone (93.7%), cefepime (95.6%), and amikacin (97.6%) [13]. A study in Brazil found that effective antibiotics against *Proteus mirabilis* isolated from patients with community-acquired urinary tract infection were as follows: sulfamethoxazole (78.1%); naphthalinic acid and gentamicin (94.5%); norfloxacin and ciprofloxacin (96.7%); amikacin and amoxicillin + clavulanic acid (99.5%); ampicillin (80.3%); cephalosporin (97.8%); cefuroxime, ceftriaxone and cefepime (98.4%); and ertapenem, meropenem, and piperacillin + tazobactam (100%) [14]. Bandy et al. retrospectively analyzed the antimicrobial spectrum of *Enterobacter* in a referral hospital in Al Khov, Saudi Arabia, in 2019 and found that the antibiotics used to treat *Proteus mirabilis* infection that were effective were as follows: furantoin (0.0%), sulfamethoxazole (15.8%), ampicillin (14.0%), cefazolin (55.8%), levofloxacin (13.1%), cefoxitin (78.2%), ceftazidime (25%), ceftriaxone (21.8%), cefepime (23.2%), amikacin (51.4%), and cefuroxime (20%) [15]. Our study found that levofloxacin had a low effective rate when used to treat *Proteus mirabilis* infection in female and male patients with urinary calculi, which is worthy of attention. The American Medical Association recommends that clinicians treat men and women who have pyelonephritis simply with fluoroquinolones (5 to 7 days) for a short period, depending on antibiotic sensitivity. Fluoroquinolones are the first line of treatment [16]. Clinical practice guidelines for the antibiotic treatment of community-acquired UTIs (CTIS) published in Korea in 2018 indicate that pyelonephritis patients who have urinary tract obstruction (e.g., urolithiasis) should be administered empiric antibiotics according to the treatment regimen of pyelonephritis alone, and fluoroquinolones can be used as early empiric antibiotics [17]. The Infectious Diseases Society of America (IDSA) clinical practice guidelines for treating simple cystitis and pyelonephritis also recommend fluoroquinolones as first-line treatment for patients with complicated pyelonephritis [18]. However, our study found that fluoroquinolone antibiotics are not suitable for the initial treatment of *Proteus mirabilis*. In view of the low response rates of *Proteus mirabilis* strains to sulfamethoxazole, levofloxacin, cefuroxime, and ceftriaxone in China, empiric antibiotic therapy for infections with *Proteus mirabilis* strains should use the more effective cefoxitin, amikacin, meropenem, imipenem, cefoperazone sulbactam, and piperacillin-tazobactam and should avoid the use of sulfamethoxazole, levofloxacin, and cefuroxime. We also assessed seasonal patterns of urine-isolated *Proteus mirabilis* sensitivity to antimicrobials. We found that the sensitivity of *Proteus mirabilis* isolates to cefuroxime and sulfamethoxazole showed a seasonal peak during winter, while levofloxacin sensitivity had a summer and winter peak. Previous studies have demonstrated a temporal association between antibiotic prescription use and enterobacterial susceptibility in the community, although we could not examine this relationship in the current study [19]. The significant seasonal changes in levofloxacin sensitivity observed here are inconsistent with previous studies of *Enterobacter* isolated from the urine of patients in Australia, where the authors reported no seasonal changes in quinolone sensitivity in subjects from Tasmania [10]. However, Australia is more restrictive in prescribing fluoroquinolones than China, which may account for the conflicting results. More recently, Martinez et al. demonstrated an association between ciprofloxacin sensitivity in community *Enterobacter* urine isolates and ciprofloxacin used during the preceding 3–6 months, suggesting that ciprofloxacin sensitivity is responsive to short-term changes in antibiotic use [20]. The seasonal variation in sulfamethoxazole sensitivity observed here is inconsistent with previous studies on the seasonal relationship between community antibiotic use and resistance in the United States. The authors suggest that sulfamethoxazole sensitivity is high during summer unlike during other seasons [20]. Therefore, further investigation is needed to understand the drivers of seasonal variations in *Proteus mirabilis* susceptibility to sulfamethoxazole.

## 5. Conclusions

Patients with *Proteus mirabilis*-infected urinary calculi in developing nations such as China are infected with strains that have a high rate of antibiotic resistance. Because of the substantial number of female patients with urolithiasis caused by *Proteus mirabilis* infection, levofloxacin, sulfamethoxazole, and cefuroxime should no longer be the primary options for empiric antibiotic therapy. Cefoxitin, a second-generation cephalosporin, and ceftazidime, a third-generation cephalosporin, should be preferred instead. We feel that primary care physicians should be taught how to choose more suitable antibiotics. Regional studies on UTIs involving urinary stones should be performed more regularly, as antibiotic resistance changes by season and year.

## Figures and Tables

**Table 1:** Antibiotic agents used for determination of the susceptibility pattern of *Proteus mirabilis*.

Component	Antibiotic
Carbapenem	Meropenem
	Imipenem
Fluoroquinolone	Levofloxacin
Second-generation cephalosporin	Cefoxitin
	Cefuroxime
Third-generation cephalosporin	Ceftazidime
	Ceftriaxone
Third-generation cephalosporin with *β*-lactamase inhibitor	Cefoperazone/Sulbactam
Fourth-generation cephalosporin	Cefepime
Aminoglycoside	Amikacin
Ampicillin with *β*-lactamase inhibitor	Piperacillin/Tazobactam
Sulfamethoxazole	Sulfamethoxazole

**Table 2:** Sex distribution of *Proteus mirabilis* urinary tract infections in patients with urinary stones (%).

Antibiotic	Total (*n* = 317)	Male (*n* = 115)	Female (*n* = 202)	*p*
Meropenem	311 (98.1)	111 (96.5)	200 (99.0)	0.257
Imipenem	305 (96.2)	108 (93.9)	197 (97.5)	0.189
Levofloxacin	183 (57.7)	56 (48.7)	127 (62.9)	0.014
Cefuroxime	161 (50.8)	53 (46.1)	108 (53.5)	0.206
Cefoxitin	271 (85.5)	93 (80.9)	178 (88.1)	0.078
Ceftazidime	276 (87.1)	98 (85.2)	178 (88.1)	0.459
Ceftriaxone	196 (61.8)	64 (55.7)	132 (65.4)	0.088
Cefoperazone/Sulbactam	304 (95.9)	109 (94.8)	195 (96.5)	0.450
Cefepime	267 (84.2)	93 (80.9)	174 (86.1)	0.178
Amikacin	314 (99.1)	114 (99.1)	200 (99.0)	0.915
Piperacillin/Tazobactam	278 (87.7)	97 (84.4)	181 (89.6)	0.171
Sulfamethoxazole	107 (33.8)	48 (41.7)	59 (29.2)	0.023

**Table 3:** Annual and total sensitivity profiles based on antibiotic sensitivity trials of *Proteus mirabilis* isolates from urinary calculus patients during the 2019–2021 study period (%).

Antibiotic	2019 (*n* = 101)	2020 (*n* = 112)	2021 (*n* = 104)	2019–2021 (*n* = 317)	*p* value
Meropenem	99 (98.0)	110 (98.2)	102 (98.1)	311 (98.1)	0.994
Imipenem	96 (95.1)	108 (96.4)	101 (97.1)	305 (96.2)	0.733
Levofloxacin	69 (68.3)	57 (50.8)	57 (54.8)	183 (57.7)	0.028
Cefuroxime	59 (58.4)	59 (52.7)	43 (41.4)	161 (50.8)	0.045
Cefoxitin	84 (83.2)	99 (88.4)	88 (84.6)	271 (85.5)	0.532
Ceftazidime	85 (84.2)	102 (91.1)	89 (85.6)	276 (87.1)	0.278
Ceftriaxone	66 (65.4)	73 (65.2)	57 (54.8)	196 (61.8)	0.198
Cefoperazone/Sulbactam	97 (96.0)	106 (94.6)	101 (97.1)	304 (95.9)	0.655
Cefepime	85 (84.2)	95 (84.8)	87 (83.7)	267 (84.2)	0.972
Amikacin	100 (98.6)	112 (100)	102 (98.1)	314 (99.1)	0.345
Piperacillin/Tazobactam	88 (87.1)	97 (86.6)	93 (89.4)	278 (87.7)	0.802
Sulfamethoxazole	32 (31.7)	43 (38.4)	32 (30.8)	107 (33.8)	0.430

**Table 4:** Antibiotic sensitivity of *Proteus mirabilis* isolated from urine of patients with urinary calculi in different seasons (%).

Antibiotic	Total (*n* = 317)	Spring (*n* = 69)	Summer (*n* = 83)	Autumn (*n* = 107)	Winter (*n* = 58)	*p* value
Meropenem	311 (98.1)	69 (100)	81 (97.6)	103 (96.3)	58 (100)	0.211
Imipenem	305 (96.2)	67 (97.1)	81 (97.6)	100 (93.5)	57 (98.3)	0.324
Levofloxacin	183 (57.7)	40 (58.0)	60 (72.3)	58 (54.2)	25 (43.1)	0.005
Cefuroxime	161 (50.8)	39 (56.5)	45 (54.2)	39 (36.5)	38 (65.5)	0.002
Cefoxitin	271 (85.5)	56 (81.2)	68 (81.9)	94 (87.9)	53 (91.4)	0.363
Ceftazidime	276 (87.1)	61 (88.4)	67 (80.7)	93 (86.9)	55 (94.8)	0.104
Ceftriaxone	196 (61.8)	48 (69.0)	52 (62.7)	56 (52.3)	40 (69.0)	0.069
Cefoperazone/Sulbactam	304 (95.9)	65 (94.2)	80 (96.4)	104 (97.2)	55 (94.8)	0.758
Cefepime	267 (84.2)	61 (88.4)	65 (78.3)	90 (84.1)	51 (87.9)	0.298
Amikacin	314 (99.1)	68 (98.6)	82 (98.8)	107 (100)	57 (98.3)	0.651
Piperacillin/Tazobactam	278 (87.7)	60 (87.0)	71 (85.5)	95 (88.8)	52 (89.7)	0.839
Sulfamethoxazole	107 (33.8)	30 (43.5)	18 (21.7)	31 (29.0)	28 (48.3)	0.002

## Data Availability

The data that support the conclusions of this study are accessible upon reasonable request from the corresponding author.
